# Disrupted theta oscillation propagation in healthy elderly individuals with apolipoprotein E epsilon 4 allele

**DOI:** 10.3389/fnins.2025.1579329

**Published:** 2025-08-06

**Authors:** Duho Sihn, Min-Ki Kim, Sung-Phil Kim

**Affiliations:** ^1^Department of Biomedical Engineering, Ulsan National Institute of Science and Technology, Ulsan, Republic of Korea; ^2^Translational Brain Research Center, Catholic Kwandong University, International St. Mary’s Hospital, Incheon, Republic of Korea

**Keywords:** Alzheimer’s disease, apolipoprotein E epsilon 4 allele, EEG, theta oscillation, traveling wave, local phase gradient

## Abstract

**Introduction:**

The apolipoprotein E epsilon 4 allele (APOE4) is one of the most influential genetic risk factors for late-onset Alzheimer’s disease (LOAD). Past studies have identified electroencephalogram (EEG) biomarkers in theta oscillations shared by both healthy individuals with APOE4 and patients with Alzheimer’s disease (AD), indicating the potentials of EEG biomarkers in healthy individuals with APOE4 for understanding and predicting LOAD. Since AD reveals a disconnection syndrome characterized by pathological synaptic connections that are also observed with APOE4, we aimed to investigate whether EEG oscillation propagation that could be influenced by impaired synaptic connections is disrupted in healthy individuals with APOE4.

**Methods:**

We analyzed a publicly available EEG dataset of elderly healthy individuals with or without APOE4. We estimated theta oscillation propagation using the local phase gradient (LPG) method and computed the median LPG length that represented the consistency of propagation directions.

**Results:**

We found that the median LPG length of frontal midline theta oscillations was smaller in participants with APOE4 than those without APOE4.

**Discussion:**

This finding indicates that APOE4 can lead to disruption in theta oscillation propagation of EEG, potentially predisposing individuals with APOE4 to LOAD.

## 1 Introduction

Alzheimer’s disease (AD) has high heritability (Bellenguez et al., 2022). Among the genetic risk factors for AD, apolipoprotein E (APOE) is recognized as one of the most influential genetic factors for late-onset AD (LOAD). LOAD specifically refers to AD that begins after 65 years old. APOE has three allele variants: epsilon 2, 3, and 4. Individuals carrying the APOE epsilon 4 allele (APOE4) have a higher likelihood of developing LOAD compared to others (Yu et al., 2014). Furthermore, healthy individuals with APOE4 exhibit brain activity patterns of electroencephalogram (EEG) similar to those seen in AD patients. Healthy individuals with APOE4 show slowing EEG frequencies (Dzianok et al., 2024), akin to what is observed in patients with AD (Cecchetti et al., 2021). Also, healthy individuals with APOE4 display an increased magnitude of theta oscillations (Ponomareva et al., 2008; Ponomareva et al., 2022), similar to patients with AD (Musaeus et al., 2018). These findings indicate the potential of EEG biomarkers in healthy individuals with APOE4 for understanding and predicting the development of LOAD.

AD is known as a disconnection syndrome (Delbeuck et al., 2003). Patients with AD exhibit pathological synaptic and axonal connections (Pereira et al., 2021; Feng et al., 2021; for a review, see Tzioras et al., 2023). EEG oscillations often propagate across the brain regions as traveling waves, which is influenced by the synaptic and axonal connections (Muller et al., 2018). As the generation of EEG oscillation propagation is based on the synaptic and axonal connections, pathological synaptic and axonal connections could lead the disruption of EEG oscillation propagation. In fact, patients with AD exhibited disrupted lagged EEG functional connectivity compared to healthy individuals (Hata et al., 2016). Since oscillation propagation can be considered a form of lagged connectivity (Muller et al., 2018), disrupted EEG oscillation propagation may also be present in AD patients. Moreover, among patients with AD, those with APOE4 exhibit more severely disrupted lagged EEG functional connectivity than other types of patients (Canuet et al., 2012).

Individuals with APOE4 have pathological synaptic connections (Yu et al., 2014), and even in healthy individuals with APOE4, pathological axonal connections are broadly observed (Chen et al., 2015), showing the possibility that EEG oscillation propagation may also be disrupted in healthy individuals with APOE4, since those structural connections are generative factors of EEG oscillation propagation (Muller et al., 2018). Moreover, these pathological structural connections in healthy individuals with APOE4 lead to dysconnectivity of blood-oxygenation-level-dependent signals (Machulda et al., 2011; Yang et al., 2014; Chen et al., 2015; Fischer et al., 2025) and also lead to abnormal EEG activity (Pedroso et al., 2022; Ponomareva et al., 2022). Consequently, we hypothesized that EEG oscillation propagation may be disrupted in healthy individuals with APOE4.

Healthy individuals with APOE4 display an increased magnitude of theta oscillations (Ponomareva et al., 2008; Ponomareva et al., 2022). While the magnitude of frontal midline theta oscillations typically decreases with healthy aging (Cummins and Finnigan, 2007; Yordanova et al., 2020), it is abnormally increased in older individuals at risk for AD (Spinelli et al., 2022). Therefore, this study focused on frontal midline theta oscillations by hypothesizing that frontal midline theta oscillation propagation would be disrupted in elderly healthy individuals with APOE4. To test this hypothesis, we analyzed a publicly available EEG dataset of elderly individuals (Dzianok and Kublik, 2024a; Dzianok and Kublik, 2024b).

Specifically, we hypothesized that temporal consistency of theta oscillation propagation, which is related to oscillatory amplitudes (Sihn and Kim, 2024), would be lower in elderly healthy individuals with APOE4 than in elderly healthy individuals without APOE4, during both resting state and task performance. Since prior studies have indicated that these lower temporal consistencies in individuals with APOE4 may be due to structural changes of neurophysiological systems including abnormal synaptic and axonal connections (Yu et al., 2014; Chen et al., 2015; Muller et al., 2018), we also assumed that these lower temporal consistencies would be observed regardless of detailed cognitive conditions, such as eyes open/closed conditions during resting state and high/low task demands during task performance.

Theta oscillation propagation was measured using the local phase gradient (LPG) method (Sihn and Kim, 2024). The LPG measures the local oscillation propagation for each EEG channel by calculating the correlation between differences in the spatial location and differences in the oscillatory phase from surrounding EEG channels. We compared the LPG of elderly healthy individuals with APOE4 to that of elderly healthy individuals without APOE4.

## 2 Materials and methods

### 2.1 Dataset

We used a publicly available dataset (Dzianok and Kublik, 2024a; Dzianok and Kublik, 2024b), accessible at https://openneuro.org/datasets/ds004796/versions/1.0.8. This dataset contains 127-channel EEG data from 79 elderly healthy participants. We divided participants into two groups: APOE4− and APOE4+. The APOE4+ group included participants with at least one APOE4 allele in a pair of alleles, while the APOE4− group consisted of those without the APOE4 allele. Table 1 summarizes the participant information.

**TABLE 1:** Participant information.

Demographic and cognitive measures	APOE4−	APOE4+	*p*-value
The number of participants	31 (15 female)	48 (25 female)	0.75 (female)
Age	54.77 ± 2.92	55.65 ± 3.23	0.24
Education level	2.71 ± 0.66	2.74 ± 0.66	0.72
Raven’s progressive matrices	53.87 ± 3.19	52.40 ± 5.28	0.34
Reaction time for the multi-source interference task (sec.); all trials	0.84 ± 0.21	0.85 ± 0.11	0.20
Reaction time for the multi-source interference task (sec.); low demand trials	0.73 ± 0.24	0.72 ± 0.093	0.30
Reaction time for the multi-source interference task (sec.); high demand trials	0.94 ± 0.19	0.98 ± 0.15	0.16
Reaction time for the Sternberg memory task (sec.); all trials	1.04 ± 0.14	1.09 ± 0.17	0.27
Reaction time for the Sternberg memory task (sec.); low demand trials	0.96 ± 0.12	0.99 ± 0.17	0.44
Reaction time for the Sternberg memory task (sec.); high demand trials	1.13 ± 0.16	1.19 ± 0.20	0.27

The APOE4+ group includes participants with at least one APOE4 allele in a pair of alleles, while the APOE4− group consists of participants without the APOE4 allele. Comparisons between the groups were conducted using a two-tailed Wilcoxon rank-sum test. The education levels consisted of values of 0, 1, 2, and 3, which indicate the primary, secondary, partial higher, and higher education, respectively.

The 127-channel EEG data were recorded via wet electrodes using the amplifier with a sampling rate of 1 kHz (actiCHamp, Brain Products GmbH, Germany). EEG recordings were obtained during three types of behavioral states. In the first resting state, participants performed eyes-open and eyes-closed tasks for 662.98 ± 40.71 s. The second state was related to adaptive cognitive control assessed through a multi-source interference task. In this task, participants selected a different digit out of three. The trials were designed under low-demand and high-demand conditions. In the low-demand trials, participants selected a digit congruent with its position, e.g., “122,” “323,” or “113.” In the high-demand trials, participants selected a digit incongruent with its position, e.g., “211,” “232,” or “331.” The third state was related to working memory assessed using the Sternberg memory task. In this task, 4 or 8 letters were presented together as a memory-set stimulus, e.g., “B C D F G H J K.” Two seconds later, three probe stimuli (single letters) were presented in succession: e.g., “Z,” “F,” or “J.” Participants were asked to respond immediately after each probe stimulus presentation whether the given probe stimulus was a part of the preceding memory-set. For more details of the behavioral tasks, see Dzianok and Kublik (2024b).

For the resting state condition, 642.48 ± 27.33 s and 642.17 ± 21.78 s of EEG data of APOE4− and APOE4+ were analyzed, respectively. The difference between the two groups was not significant (two-tailed Wilcoxon rank-sum test, *p* = 0.76). Among these, eye-open EEG data were 304.67 ± 119.20 s and 306.87 ± 127.11 s for APOE4− and APOE4+, respectively. Eye-closed EEG data were 361.27 ± 0.0069 s and 361.29 ± 0.083 s for APOE4− and APOE4+, respectively. In the resting state condition, analyses were performed across both eyes open and closed. The resting state data was analyzed as continuous data without segmentation.

For the multi-source interference task, 140.53 ± 34.60 s and 142.45 ± 18.30 s of EEG data of APOE4− and APOE4+ were analyzed, respectively. The difference between the two groups was not significant (two-tailed Wilcoxon rank-sum test, *p* = 0.21). These correspond to 165.81 ± 1.078 and 166 ± 0 trials. Among these, low demands EEG data were 61.47 ± 19.60 s and 60.58 ± 7.79 s for APOE4− and APOE4+, respectively. These correspond to 83 ± 0.82 and 83.15 ± 0.71 trials. High demands EEG data were 79.07 ± 15.64 s and 81.88 ± 12.03 s for APOE4− and APOE4+, respectively. These correspond to 82.81 ± 1.014 and 82.85 ± 0.71 trials. In the multi-source interference task, analyses were performed across all levels of task demand. In the multi-source interference task, all trials were used in the analysis. Each trial was defined from the stimulus onset to the behavioral timing, which spanned 0.84 ± 0.16 s across subjects. Reaction times were in Table 1.

For the Sternberg memory task, 591.76 ± 8.10 s and 595.56 ± 11.01 s of EEG data of APOE4− and APOE4+ were analyzed, respectively. The difference between the two groups was not significant (two-tailed Wilcoxon rank-sum test, *p* = 0.21). These correspond to 48 ± 0 and 48.04 ± 0.29 trials. Among these, low demands EEG data were 293.46 ± 3.25 s and 296.11 ± 5.18 s for APOE4− and APOE4+, respectively. These correspond to 24 ± 0 and 24.02 ± 0.15 trials. High demands EEG data were 298.30 ± 5.89 s and 299.52 ± 6.82 s for APOE4− and APOE4+, respectively. These correspond to 24 ± 0 and 24.02 ± 0.15 trials. In the Sternberg memory task, analyses were performed across all levels of task demand. In the Sternberg memory task, all trials were used in the analysis. Each trial was defined from the stimulus onset to the last behavioral timing, which spanned 12.36 ± 0.20 s across subjects. Reaction times were in Table 1.

### 2.2 EEG processing

To perform EEG preprocessing, the raw data were bandpass filtered between 0.5 and 30 Hz using a finite impulse response (FIR) filter with a 14-s order. The filtered data were then downsampled to 500 Hz. Potential EEG artifacts were removed by applying the Artifact Subspace Reconstruction procedure to the downsampled data. There was no channel that was interpolated or removed.

The preprocessed data underwent surface Laplacian filtering to mitigate the volume conduction effect. The surface Laplacian filtering was performed using the CSD toolbox (Kayser and Tenke, 2006a; Kayser and Tenke, 2006b; Kayser, 2009). The model parameters were set to *m* = 4 and λ = 10^−5^, which are common settings in related EEG oscillation studies (Tenke et al., 2011; Sihn et al., 2023; Sihn and Kim, 2024).

**FIGURE 1:**
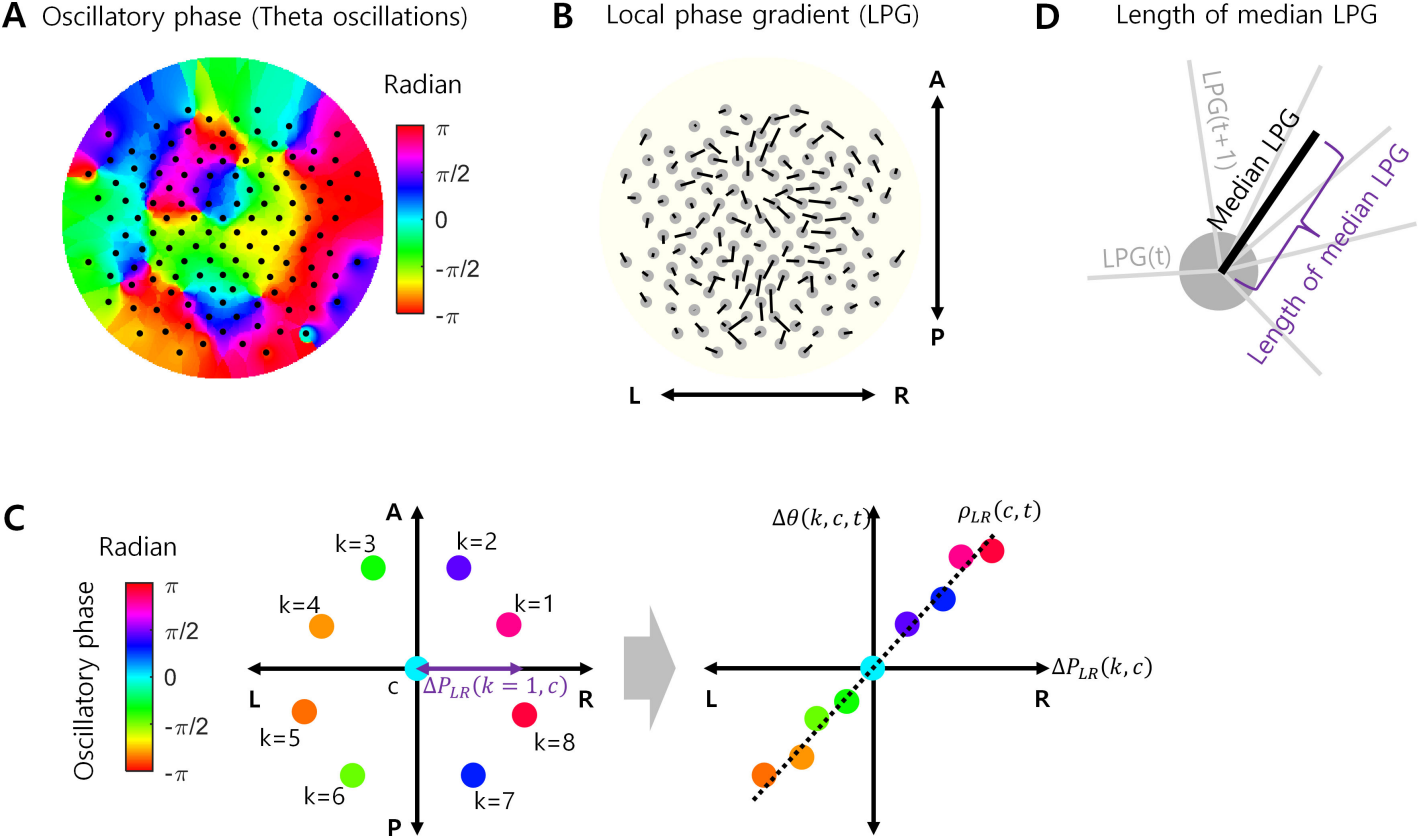
Local phase gradient (LPG). **(A)** Example of oscillatory phases in theta oscillations at every EEG channel (127 channels). **(B)** LPG calculated from the phase data in **(A)**. Originally, LPG is a 3-dimensional vector, but projected onto a 2-dimensional plane here for visualization. L, R, A, and *P* indicate left, right, anterior, and posterior, respectively. **(C)** A conceptual example of calculating LPG. For a certain channel c, the oscillatory phases of eight neighboring channels (*k* = 1, …, 8) were measured (left). For each axis passing through the location of channel c (LR axis in this example), a correlation [ρ_*XY*_(*c*,*t*)] between the projected positions of *k* i.e., [Δ*P*_*XY*_(*k*,*c*)] channels and the corresponding phase differences [Δθ(*k*,*c*,*t*)] was calculated (right). **(D)** The median LPG is the median value of LPGs over time during task performance, which is also a 3-dimensional vector. The length of the median LPG is the Euclidean norm of this vector.

After surface Laplacian filtering, the data were bandpass filtered between 4 and 7 Hz using an FIR filter with a 1.75-s order to isolate the theta oscillations. The theta oscillation data then underwent the Hilbert transform to extract the oscillatory phase for every channel (Figure 1A). Following the Hilbert transform, the phase data were downsampled to 100 Hz to increase data processing speed while containing more than 10 data points per one cycle of theta oscillations.

For the magnitude of theta oscillations, the magnitude was obtained by taking the absolute value of the Hilbert transformed data derived in the above paragraph.

### 2.3 Local phase gradient

To measure the EEG oscillation propagation, we used LPG (Sihn and Kim, 2024). Let *u*_*LR*_, *u*_*AP*_, and *u*_*SI*_ be the unit vectors corresponding to the left-right, anterior-posterior, and superior-inferior axes, respectively. Let *c* index EEG channel and *k* does a neighboring EEG channel of *c*. Let Δ*P*_*XY*_(*k*,*c*) denote a difference in spatial positions between channel *k* and channel *c* along the axis *XY*, where *XY* ∈ {*LR*,*AP*,*SI*}. LR refers to the left-right axis, AP does the anterior-posterior axis, and SI does the superior-inferior axis. Let Δθ(*k*,*c*,*t*) denote a difference in oscillatory phases between *k* and *c* at time *t*. Then, ρ_*XY*_(*c*,*t*) represents a Pearson correlation between Δ*P*_*XY*_(*k*,*c*) and Δθ(*k*,*c*,*t*) over *k* (Figure 1C). In this study, the number of neighboring channels was set to 8. From these correlations, the LPG of channel *c* at time *t* is given by Figures 1B, C:


(1) $$\begin{array}{cc} L\,P\,G\,\left(c,t\right)=\rho_{L\,R}\,\left(c,t\right)\,u_{L\,R}+\rho_{A\,P}\,\left(c,t\right)\,u_{A\,P}+\rho_{S\,I}\,\left(c,t\right)\,u_{S\,I}. & \end{array}$$

That is, a *LPG*(*c*,*t*) is a 3-dimensional vector. If oscillation propagation on the neighboring channels consistently points toward a certain direction, the length of *LPG*(*c*,*t*) would be large. The length of *LPG*(*c*,*t*) is given by the Euclidean norm of *LPG*(*c*,*t*):


(2) $$\begin{array}{cc} \mathrm{Length}\,\mathrm{of}\,L\,P\,G\,\left(c,t\right)={||L\,P\,G\,\left(c,t\right)||}_{2} & \end{array}$$

where |⋅|_2_ is an Euclidean norm.

In our analysis, we calculate the median vector of *LPG*(*c*,*t*) over *t* such that:


(3) median⁢L⁢P⁢G⁢(c)=

median{LPG(c,t)|tistimesatisfyingcertainconditions.}.

Note that median *LPG*(*c*) is also a 3-dimensional vector. If *LPG*(*c*,*t*) consistently points toward a certain direction over *t*, the length of median *LPG*(*c*) would be relatively large. The length of median *LPG*(*c*) is given by the Euclidean norm of median *LPG*(*c*) (Figure 1D):


(4) $$\begin{array}{cc} \mathrm{Length}\,\mathrm{of}\,\mathrm{median}\,L\,P\,G\,\left(c\right)={||\mathrm{median}\,L\,P\,G\,\left(c\right)||}_{2} & \end{array}$$

where |⋅|_2_ is an Euclidean norm.

If a theta oscillation propagates over a local region in a particular direction, the phase of theta oscillations measured at local channels would systematically change along the direction during the occurrence of propagation. It would result in high spatial correlations in Equations (1, 3) as well as the greater length of medial LPG in Equation (4) that represents the temporal correlation of oscillation propagation. If propagation is disrupted, it would shrink the length of median LPG by two possible ways. First, an instantaneous LPG, *LPG*(c,t), could be shortened due to lowered correlations (ρ_*XY*_(c,t)). It indicates that the local spatial consistency of propagation directionality reduces. Second, the direction of *LPG*(c,t) could be more variable over t, which indicates that the temporal consistency of propagation directionality reduces.

### 2.4 Statistical tests

For demographic, cognitive, and behavioral performance measures comparisons between APOE4− and APOE4+, two-tailed rank-sum test was utilized with the statistical significance level of *p*-value 0.05. For comparisons of analyzed data length between APOE4− and APOE4+, two-tailed rank-sum test was utilized with the statistical significance level of *p*-value 0.05.

For statistical comparisons, we focused only on the frontal midline 8 channels according to our hypothesis. Those channels were Fz, AFz, F1, F2, AFF1h, FFC1h, FFC2h, and AFF2h. In the spatial consistency comparisons, for each EEG channel, we tested whether “the length of LPG (see, Equation 2)” in the APOE4+ group was significantly smaller than that in the APOE4− group using a one-tailed Wilcoxon rank-sum test, based on the hypothesized disruption of propagation by APOE4. Significance was determined at a false discovery rate (FDR)-corrected *p*-value of 0.05. FDR correction was applied across frontal midline 8 channels. For FDR corrections, we adopted the Benjamini and Hochberg procedure. In temporal consistency comparisons, they were similar to the spatial consistency comparisons, but we used “the length of the median LPG (see, Equation 4)” instead of “the length of LPG” for differentiation.

To assess how well we could distinguish between APOE4+ and APOE4− participants, we averaged the lengths of the median LPGs from EEG channels that showed significant differences between the two groups. This significant-channel-averaged length of the median LPG was then compared between APOE4+ and APOE4− using a one-tailed Wilcoxon rank-sum test. Significance was determined at a *p*-value of 0.05.

To compare the magnitude of theta oscillations in APOE4− and APOE4+ groups, we utilized two-tailed Wilcoxon rank-sum test. Significance was determined at a false discovery rate (FDR)-corrected *p*-value of 0.05. FDR correction was applied across frontal midline 8 channels. For FDR corrections, we adopted the Benjamini and Hochberg procedure.

## 3 Results

According to our hypothesis, we focused only on the frontal midline 8 channels. Those channels were Fz, AFz, F1, F2, AFF1h, FFC1h, FFC2h, and AFF2h. There was no significant difference of reaction times of APOE4− and APOE4+ in both the multi-source interference task and the Sternberg memory task (Table 1).

We measured the local spatial consistency of theta oscillation propagation directionality using the length of LPG (see Equation 2). The length of LPG at each time instant was summarized by the median value of ||*LPG*(*c*,*t*)||_2_ over *t* for each channel in each participant. Note that this median value was different from the length of median LPG defined in Equation (4). During the resting state, the LPG length of the APOE4+ group was not significantly different from that of the APOE4− group at every channel (*c*) (one-tailed Wilcoxon rank-sum test, FDR-corrected *p* < 0.05). During the multi-source interference task, the LPG length of the APOE4+ group was significantly smaller than that of the APOE4− group at only one channel out of 8 (@AFF2h, one-tailed Wilcoxon rank-sum test, FDR-corrected *p* < 0.05). During the Sternberg memory task, the LPG length of the APOE4+ group was also not significantly different from that of the APOE4− group at every channel (one-tailed Wilcoxon rank-sum test, FDR-corrected *p* < 0.05). This result indicated that the local spatial consistency of theta oscillation propagation did not change in elderly healthy individuals with APOE4 compared to those without APOE4.

**FIGURE 2:**
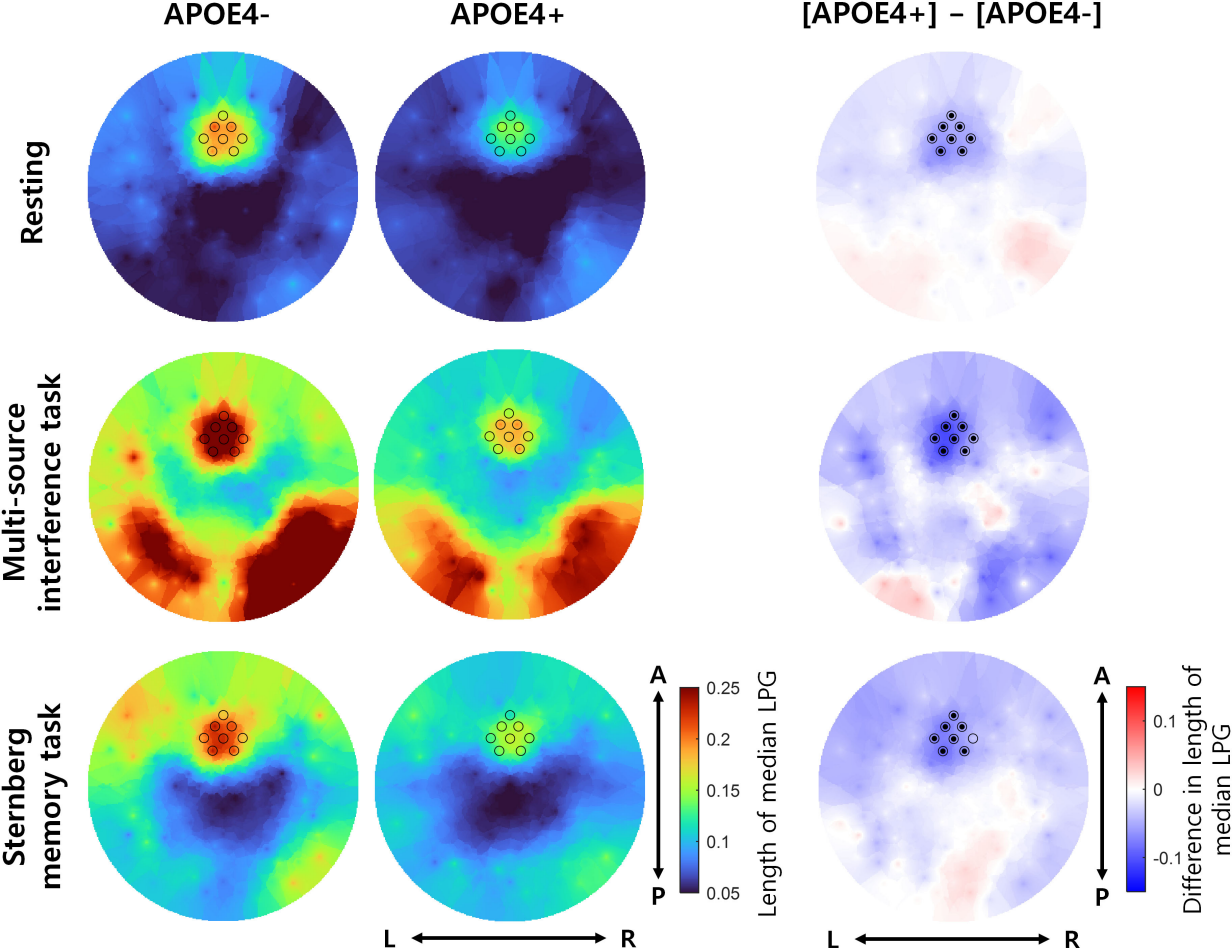
The temporal consistency of theta oscillation propagation directionality in participants with or without APOE4. The left and middle columns represent the length of median LPG in the APOE4– and APOE4+ groups, respectively. Each black circle indicates the position of an analyzed EEG channel. The right column shows the difference in the length of median LPG between the APOE4+ and APOE4– groups. Black dots indicate EEG channels where the length of median LPG in the APOE4+ group is significantly smaller than that in the APOE4– group (one-tailed Wilcoxon rank-sum test, FDR-corrected *p* < 0.05). L, R, A, and *P* indicate left, right, anterior, and posterior, respectively.

Next, we investigated whether the temporal consistency of theta oscillation propagation directionality reduced in the APOE4+ group. To measure the temporal consistency of theta oscillation propagation directionality, we used the length of median LPG (see Equation 4). During the resting state, the length of median LPG of the APOE4+ group was significantly smaller than that of the APOE4− group at 8 out of 8 channels (one-tailed Wilcoxon rank-sum test, FDR-corrected *p* < 0.05). During the multi-source interference task, the length of median LPG of the APOE4+ group was significantly smaller than of the APOE4− group at 8 out of 8 channels (one-tailed Wilcoxon rank-sum test, FDR-corrected *p* < 0.05). During the Sternberg memory task, the length of median LPG of the APOE4+ group was significantly smaller than that of the APOE4− group at 7 out of 8 channels (one-tailed Wilcoxon rank-sum test, FDR-corrected *p* < 0.05) (Figure 2). Specific test statistics and *p*-values were in Table 2. This result indicates that theta oscillation propagation in the frontal midline region was disrupted in elderly healthy individuals with APOE4, mainly due to temporal inconsistency of propagation directionality.

**TABLE 2:** Test statistics and FDR-corrected *p*-values for statistical comparisons of the temporal consistency.

	EEG channels							
Conditions	Fz	AFz	F1	F2	AFF1h	FFC1h	FFC2h	AFF2h
Resting (test statistics)	1,545	1,489	1,525	1,513	1,530	1,584	1,492	1,472
Resting (FDR-corrected *p*-values)	0.0043	0.0072	0.0043	0.0050	0.0043	0.0023	0.0072	0.010
Multi-source interference task (test statistics)	1,629	1,488	1,570	1,448	1,548	1,612	1,520	1,467
Multi-source interference task (FDR-corrected *p*-values)	3.83 ×10^–4^	0.0086	0.0013	0.019	0.0020	7.44 ×10^–4^	0.0040	3.83 ×10^–4^
Sternberg memory task (test statistics)	1,533	1,455	1,551	1,363	1,595	1,701	1,532	1,507
Sternberg memory task (FDR-corrected *p*-values)	0.0027	0.018	0.0024	0.11	7.44 ×10^–4^	1.51 ×10^–5^	0.0027	0.0050

**FIGURE 3:**
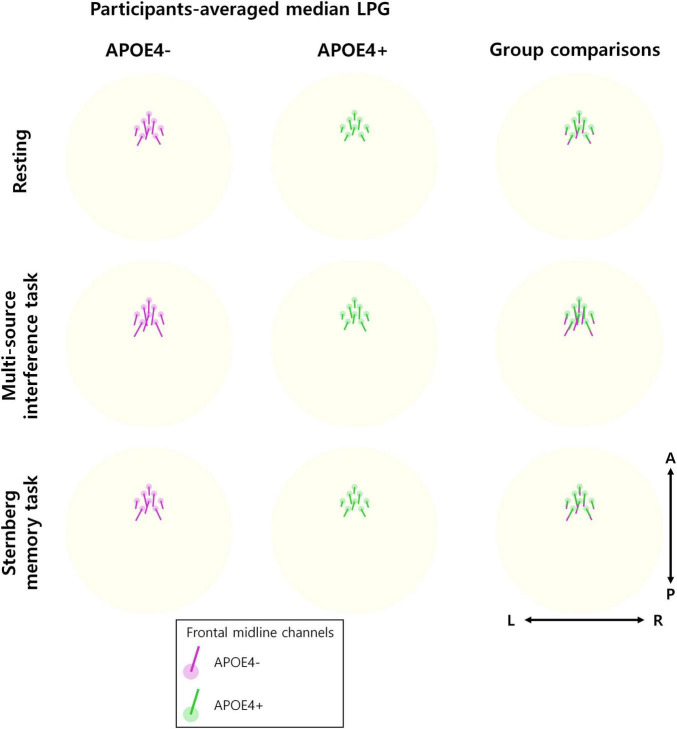
Direction of theta oscillation propagation. The participant-averaged median LPG (line) is displayed at eight frontal midline channels (circle) for each of the APOE4– group (left) and the APOE4+ group (middle), respectively. The directions of the participant-averaged median LPG are similar between the groups. This can be observed in the overwriting LPGs of two groups (right). The 3D median LPG is projected onto a 2D plane for visualization.

Furthermore, we found that the direction of median LPG appeared to be similar between the APOE4− and APOE4+ groups (Figure 3). Therefore, APOE4 may disrupt the temporal consistency of frontal theta oscillation propagation directionality rather than the propagation direction itself.

**FIGURE 4:**
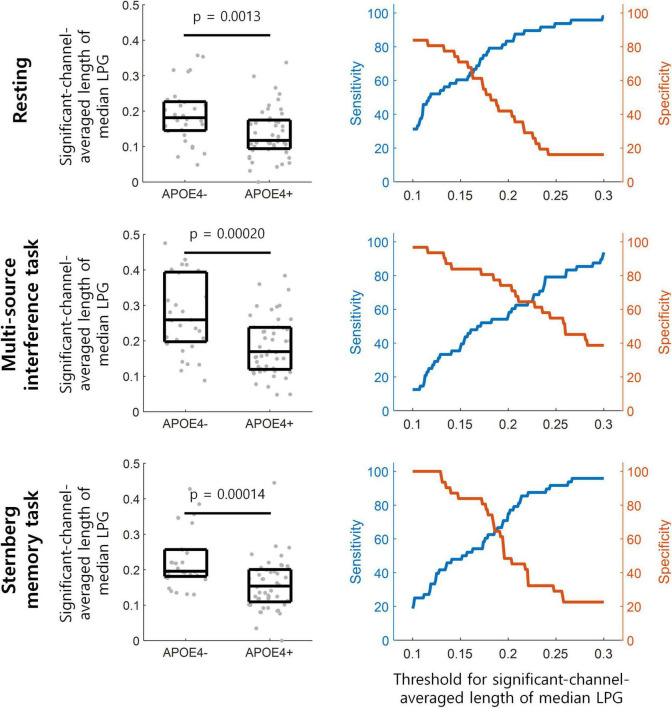
Detection of participants with APOE4 by theta oscillation propagation. (Left) The length of median LPG is averaged over frontal midline EEG channels in each participant denoted by gray dots. This averaged length of median LPG is significantly smaller in the APOE4+ than in the APOE4– groups (one-tailed Wilcoxon rank-sum test). The three horizontal lines within each black box indicate the 25th, 50th, and 75th percentiles of the data, respectively. (Right) The performance of distinguishing participants with APOE4 from those without APOE4 at various thresholds for the channel-averaged length of median LPG.

To assess the discrimination ability between the APOE4+ and APOE4− groups using theta oscillation propagation, we averaged the lengths of median LPG from eight frontal midline EEG channels (Fz, AFz, F1, F2, AFF1h, FFC1h, FFC2h, and AFF2h). This channel-averaged length of median LPG was then compared between the APOE4+ and APOE4− groups under each experimental condition. For all conditions—resting, multi-source interference task, and Sternberg memory task—, the channel-averaged length of median LPG was smaller in the APOE4+ than in the APOE4− groups (Figure 4 left) (one-tailed Wilcoxon rank-sum test, *p*s < 0.002).

During the resting state, APOE4+ could be distinguished from APOE4− at the channel-averaged length of median LPG of 0.139, with sensitivity of 58.33% and specificity of 77.42%. During the multi-source interference task, APOE4+ could be distinguished from APOE4− at 0.239, with sensitivity of 79.17% and specificity of 58.06%. During the Sternberg memory task, APOE4+ could be distinguished from APOE4− at 0.128, with sensitivity of 41.67% and specificity of 100% (Figure 4 right).

**FIGURE 5:**
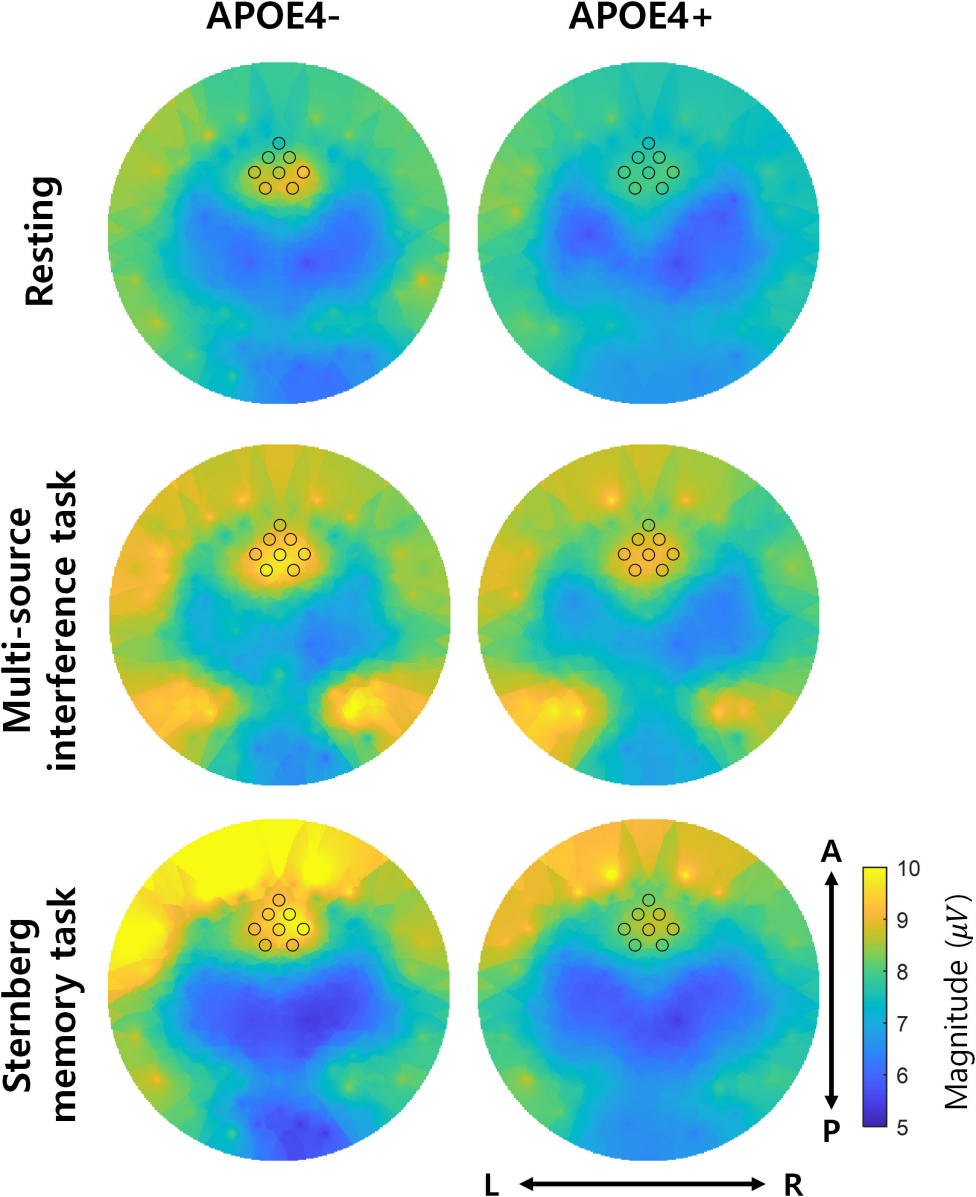
Magnitude of theta oscillations. The left and right columns represent the magnitude of theta oscillations in the APOE4– and APOE4+ groups, respectively. Each black circle indicates the position of EEG channels analyzed in the study. L, R, A, and *P* indicate left, right, anterior, and posterior, respectively. Note that the two groups did not significantly differ in magnitude of theta oscillations (two-tailed Wilcoxon rank-sum test, FDR-corrected *p* < 0.05).

Since it is known that the temporal consistency tends to correlate with the magnitude of oscillations, we further investigated the magnitude of theta oscillations of APOE4− and APOE4+ groups. Overall, frontal midline theta oscillations tended to have a larger magnitude than other regions in APOE4−. Moreover, these large magnitudes tended to decrease in APOE4+ group, but it is not statistically significant (two-tailed Wilcoxon rank-sum test, FDR-corrected *p* < 0.05) (Figure 5).

Since EEG oscillation propagation is related to information transmission, we further investigated whether the disruption of theta oscillation propagation in the APOE4+ group could predict individual variation in cognitive processing. To this end, we analyzed a relationship between the channel-averaged length of median LPG and cognitive performance. Specifically, we utilized the cognitive flexibility performance metrics from Raven’s progressive matrices. The reason why we choose the “Raven’s progressive matrices” is that this metric represents the cognitive flexibility which is also related to the multi-source interference task.

Group-wise behavioral assessment showed that the cognitive flexibility of the APOE4+ group was not significantly different from those of the APOE4− group (Table 1). During the multi-source interference task, which is associated with cognitive flexibility, the channel-averaged length of median LPG in individual participants was not significantly correlated with the cognitive flexibility performance in both groups (Pearson correlation, rho = 0.048 and −0.23 in APOE4+, *p* = 0.80 and 0.11 in APOE4−).

## 4 Discussion

We analyzed EEG theta oscillation propagation using the LPG method (Sihn and Kim, 2024) and examined whether the length of median LPG, which represents the consistency of oscillation propagation directionality, was smaller in APOE4+ participants compared to APOE4− participants. We found that the median LPG length of frontal midline theta oscillations was smaller in APOE4+ participants, indicating disrupted theta oscillation propagation in this group. This finding confirmed our hypothesis that frontal midline theta oscillation propagation would be disrupted in elderly healthy individuals with APOE4, which suggests that APOE4 may contribute to disconnections between brain regions, potentially predisposing individuals to LOAD.

Theta oscillations are closely associated with the encoding and retrieval of information, playing a crucial role in coordinating communication between different brain regions (for a review, Hasselmo and Stern, 2014). The propagation of theta oscillations is believed to enhance the synchronization of neuronal firing, thereby improving the efficiency of information transfer across neural circuits (Kragel et al., 2020). The disrupted theta oscillation propagation found in this study may exhibit the disrupted efficiency of information transfer within the frontal midline region in the APOE4+ group.

Since the magnitude of frontal midline theta oscillations are generally associated with cognitive performance (Zhang et al., 2016), we suspect that the disruption of theta oscillation propagation in the frontal midline region might be related to changes in cognitive performance by potentially affecting neural information transmission. In general, brain oscillation propagation is known to play a role in information transmission in many EEG (Alamia and VanRullen, 2019; Pang et al., 2020; Alamia et al., 2020; Alamia et al., 2023) and electrocorticogram (ECoG) studies (Zhang et al., 2018; Stolk et al., 2019; Halgren et al., 2019; Kleen et al., 2021; Mohan et al., 2024). From the group-wise analysis, however, there was no significant difference in cognitive performance between APOE4+ and APOE4− participants. From the individual analysis, when we related the degree of theta oscillation propagation using the length of median LPG to cognitive performance, both APOE4+ and APOE4− groups did not exhibit correlations between the length of median LPG and the cognitive flexibility performance. These limited relationships between theta oscillation propagation and cognitive performance suggest that the role of theta oscillatory propagation in information transmission in elderly healthy individuals still needs further exploration.

The cognitive normality of individuals in the dataset we used is based on the observation of experimenters and the description of participants themselves (Dzianok and Kublik, 2024b), rather than depending on a global cognitive screener such as the mini-mental state examination (Folstein et al., 1975) or the Montreal cognitive assessment (Nasreddine et al., 2005). This leads relatively vulnerable information for the cognitive normality of individuals, and it is a limitation of this study. Future studies may replicate the results of this study with a global cognitive screener. In task performance conditions, we used all trials regardless of correctness. This was due to the lack of correctness information in the dataset we used. However, it may be more appropriate to analyze only the trials with correct behavior. Future studies may replicate the analysis of this study containing only correct trials. Patients with AD exhibit reduced EEG functional connectivity (Hata et al., 2016) and those carrying APOE4 show more severe disruption of connectivity (Canuet et al., 2012). Since oscillation propagation can reflect time-lagged functional connectivity (Muller et al., 2018), our finding of disrupted EEG theta oscillation propagation in healthy participants with APOE4 set up a hypothesis that this propagation would also be disrupted in AD patients. Yet, this hypothesis has not been confirmed in patients with LOAD in the present study, which must be addressed in the follow-up studies. In addition, the observation of disrupted EEG theta oscillation propagation does not assure that EEG theta oscillation propagation may be a predictor of the development of LOAD. Longitudinal neurophysiological studies will be needed to verify the possibility of using EEG theta oscillation propagation as a predictor of LOAD. Lastly, another limitation lies at the lack of a model explaining the relationship of EEG theta oscillatory propagation with the presence of APOE4. Future research should focus on building more comprehensive models by leveraging diverse genetic data and a wider range of behavioral experimental results.

Healthy individuals with the APOE4 allele exhibit several EEG biomarkers similar to those seen in AD patients (Dzianok et al., 2024, Ponomareva et al., 2008; Ponomareva et al., 2022). Therefore, identifying EEG biomarkers in healthy individuals with APOE4 is crucial for predicting the development of LOAD and providing preventive care. This study suggests a new EEG biomarker in healthy individuals with APOE4 as the disruption of frontal midline theta oscillation propagation. Our finding may pave the way of using EEG oscillation propagation for the diagnosis of AD.

## 5 Conclusion

We observed that the median LPG length of frontal midline theta oscillations was smaller in APOE4+ participants compared to APOE4− participants, indicating disrupted theta oscillation propagation in APOE4+. This disruption in theta oscillation propagation suggests that APOE4 may contribute to disconnections between brain regions, potentially predisposing individuals to LOAD and leading to disrupted theta oscillation propagation.

## Data Availability

The original contributions presented in this study are included in this article/supplementary material, further inquiries can be directed to the corresponding author.
